# Chloramphenicol Mediates Superoxide Production in Photosystem II and Enhances Its Photodamage in Isolated Membrane Particles

**DOI:** 10.3389/fpls.2016.00479

**Published:** 2016-04-08

**Authors:** Ateeq Ur Rehman, Sandeesha Kodru, Imre Vass

**Affiliations:** Institute of Plant Biology, Biological Research Centre of the Hungarian Academy of SciencesSzeged, Hungary

**Keywords:** photoinhibition, Photosystem II, chloramphenicol, superoxide

## Abstract

Chloramphenicol (CAP) is an inhibitor of protein synthesis, which is frequently used to decouple photodamage and protein synthesis dependent repair of Photosystem II during the process of photoinhibition. It has been reported earlier that CAP is able to mediate superoxide production by transferring electrons from the acceptor side of Photosystem I to oxygen. Here we investigated the interaction of CAP with Photosystem II electron transport processes by oxygen uptake and variable chlorophyll fluorescence measurements. Our data show that CAP can accept electrons at the acceptor side of Photosystem II, most likely from Pheophytin, and deliver them to molecular oxygen leading to superoxide production. In addition, the presence of CAP enhances photodamage of Photosystem II electron transport in isolated membrane particles, which effect is reversible by superoxide dismutase. It is concluded that CAP acts as electron acceptor in Photosystem II and mediates its superoxide dependent photodamage. This effect has potential implications for the application of CAP in photoinhibitory studies in intact systems.

## Introduction

Photosynthesis is a process in which green plants, algae and cyanobacteria utilize energy from sunlight to manufacture carbohydrates from carbon dioxide and water. This process is the ultimate source of energy for all plants to drive their metabolic processes. Too much light reaching the photosynthetic apparatus can cause photodamage and ultimately can lead to the death of a cell. This stress situation is known as photoinhibition (Arntzen et al., 1984; Aro et al., 1993; Vass and Aro, 2008). The major damage of the photosynthetic apparatus under high light conditions is impairment of electron transport in the Photosystem II (PSII) complex, as well as damage of the D1 reaction center subunit (Ohad et al., 1984; Prasil et al., 1992). Important mediators of photodamage in plant cells are the various reactive oxygen species (ROS), such as singlet excited oxygen, free radicals (superoxide and hydroxyl ions) and peroxides, which are produced mainly in the chloroplasts and mitochondria (Apel and Hirt, 2004). The activity of the photodamaged PSII complex can be restored via the so called PSII repair cycle in which *de novo* synthesis of the damaged D1 subunits plays a key role (Aro et al., 1993; Baena-Gonzalez and Aro, 2002; Komenda et al., 2007; Nixon et al., 2010).

Light stress to PSII becomes a problem for photosynthetic capacity when the rate of photodamage exceeds the capacity of repair processes. Therefore, it is important to monitor separately the rates of photodamage and of the protein synthesis dependent repair. Decoupling of photodamage and repair can be achieved by protein synthesis inhibitors, such as lincomycin or chloramphenicol (CAP), which inhibit translation elongation in chloroplasts (Mulo et al., 2003; Chow et al., 2005; Tikkanen et al., 2014) or in cyanobacterial cells (Constant et al., 1997; Nishiyama et al., 2001, 2005; Sicora et al., 2003; Takahashi and Murata, 2005; Takahashi et al., 2009). While there are no reports concerning the participation of lincomycin in photosynthetic electron transport, CAP has been reported to accept electrons from the acceptor side of Photosystem I and to transfer them to molecular oxygen leading to superoxide production (Okada et al., 1991). Superoxide radicals have high reactivity, therefore, it is expected that locally generated superoxide will induce damaging effects in the vicinity of its production. This finding has been considered as a source of potential artifact by several research groups, who used lincomycin instead of CAP in photoinhibition studies (Tyystjarvi and Aro, 1996; Constant et al., 1997; Tyystjarvi et al., 2002; Chow et al., 2005; Campbell and Tyystjärvi, 2012; Miyata et al., 2012; Tikkanen et al., 2014). However, other groups kept using CAP in measurements of PSII photodamage (Nishiyama et al., 2001, 2005; Takahashi and Murata, 2005; Takahashi et al., 2009).

In the present work we investigated whether CAP has the capacity to interact directly with PSII electron transport in isolated membrane particles. Our data show that CAP acts as an electron acceptor to PSII and mediates superoxide production, which enhances photodamage of PSII.

## Materials and Methods

### PSII Membrane Preparation

Photosystem II membrane particles were isolated from fresh spinach leaves as described earlier (Vass et al., 1987) and suspended in buffers containing 40 mM MES-NaOH (pH 6.5), 15 mM MgCl_2_, 15mM CaCl_2_ and 1 M betaine, respectively. PSII membranes were stored in -80°C for further use.

### Light Induced Oxygen Uptake Measurements

O_2_ uptake rates in PSII particles were measured by using a Hansatech DW2 O_2_ electrode at 4°C under illumination with 500 μmole m^-2^s^-1^ light intensity. The total duration of illumination was 1 min. DCMU, which blocks electron transport at the Q_B_ site of PSII was also added at a concentration of 10 μM when indicated. In order to confirm superoxide formation the rate of oxygen uptake was also measured in the presence of 20 units/mg superoxide dismutase (SOD) that converts 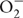 partly back to O_2_, as well as after addition of 1000 units of bovine serum catalase that converts H_2_O_2_, which is produced by SOD from 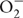, to H_2_O and O_2_. One mililiter aliquot of PSII membrane particles at 5 μg Chl mL^-1^ concentration was used in each measurement.

### Photoinhibitory Treatment

The PSII particles were resuspended at 5 μg Chl mL^-1^ in 40 mL volume and illuminated with 500 μmole m^-2^s^-1^ light intensity in the presence and absence of CAP (200 μg/mL). The temperature during illumination was maintained at 4°C. The samples were also illuminated in the presence of SOD (20 units mg^-1^). For monitoring PSII activity the rate of O_2_ evolution was measured at the indicated time points. Photosynthetic activity of irradiated PSII membranes was also assessed by measuring the so called OJIP transient of variable Chl fluorescence during application of a 2 s saturating pulse (Strasser et al., 1995) by using an FL-3000 fluorometer (PSI). F_v_/F_m_ was obtained by calculating (F_m_-F_o_)/F_m_, where F_o_ and F_m_ represent the minimum fluorescence in dark adapted sample, and the maximal fluorescence yield under continuous saturating light, respectively.

## Results and Discussion

### CAP Acts as Electron Acceptor in PSII

**FIGURE 1:**
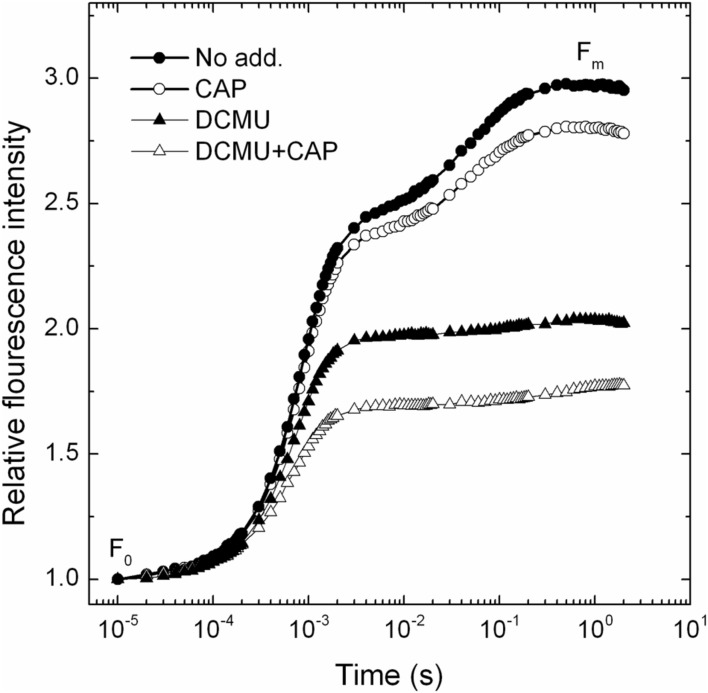
**The effect of CAP on PSII activity in PSII membrane particles as quantified by fast chlorophyll fluorescence rise.** Photosynthetic activity of spinach PSII membranes was assessed by measuring OJIP transients of chlorophyll fluorescence. The experiments were performed in the absence (closed symbols) and presence of chloramphenicol (open symbols) without further addition (circles) or in the presence of 10 μM DCMU (triangles). The curves are shown after normalization to the same Fo values.

Chloramphenicol has been reported earlier to take up electrons at the acceptor side of PSI (Okada et al., 1991). In order to check if similar phenomenon occurs in PSII, or not, the so called OJIP Chl fluorescence transient was measured in the absence and presence of CAP. As shown in **Figure 1** the maximal fluorescence level (F_m_) was decreased in the presence of CAP (open circles), which is consistent with the presence of an electron acceptor that prevents complete reduction of the Q_A_ primary quinone electron acceptor. In order to verify if CAP takes up electrons before or after the Q_B_ binding site we used DCMU, which inhibits electron transport from 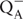 to Q_B_ by preventing PQ binding to the Q_B_ site. Interestingly CAP induced decrease of the F_m_ level also in the presence of DCMU (**Figure 1**, open triangles), which indicates that CAP takes up electrons from PSII before the DCMU block, i.e., either directly from 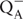 or from Phe^-^. Considering the very negative redox potential of CAP, E_m_(CAP/CAP^-^) = -543 mV (Kapoor and Varshney, 1997), the efficiency of electron transfer from 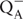 (E_m_(Q_A_/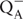) = -120 to -140 mV, Shibamoto et al., 2009) to CAP should be very low. On the other hand the redox potential of Phe [E_m_(Phe/Pheo^-^) = -505 to -535 mV (Shibamoto et al., 2009; Allakhverdiev et al., 2010)] allows energetically efficient interaction with Phe^-^. Therefore, although the lifetime of Phe^-^ is very short (ca. 200 ps) it is a possible candidate to act as an electron donor for the reduction of CAP. This finding is in agreement with previous suggestions that Phe^-^ can act as direct electron donor to O_2_ and can support superoxide production (Pospísil, 2012).

### CAP Induces Superoxide Production in Isolated PSII Particles

It has been reported previously (Okada et al., 1991) that CAP mediates superoxide production in thylakoids by transferring electrons from the PSI acceptor side to oxygen. Since we have shown that CAP functions not only as PSI electron acceptor, but takes up electrons also from PSII it has a potential to produce superoxide in PSII complexes as, well.

**FIGURE 2:**
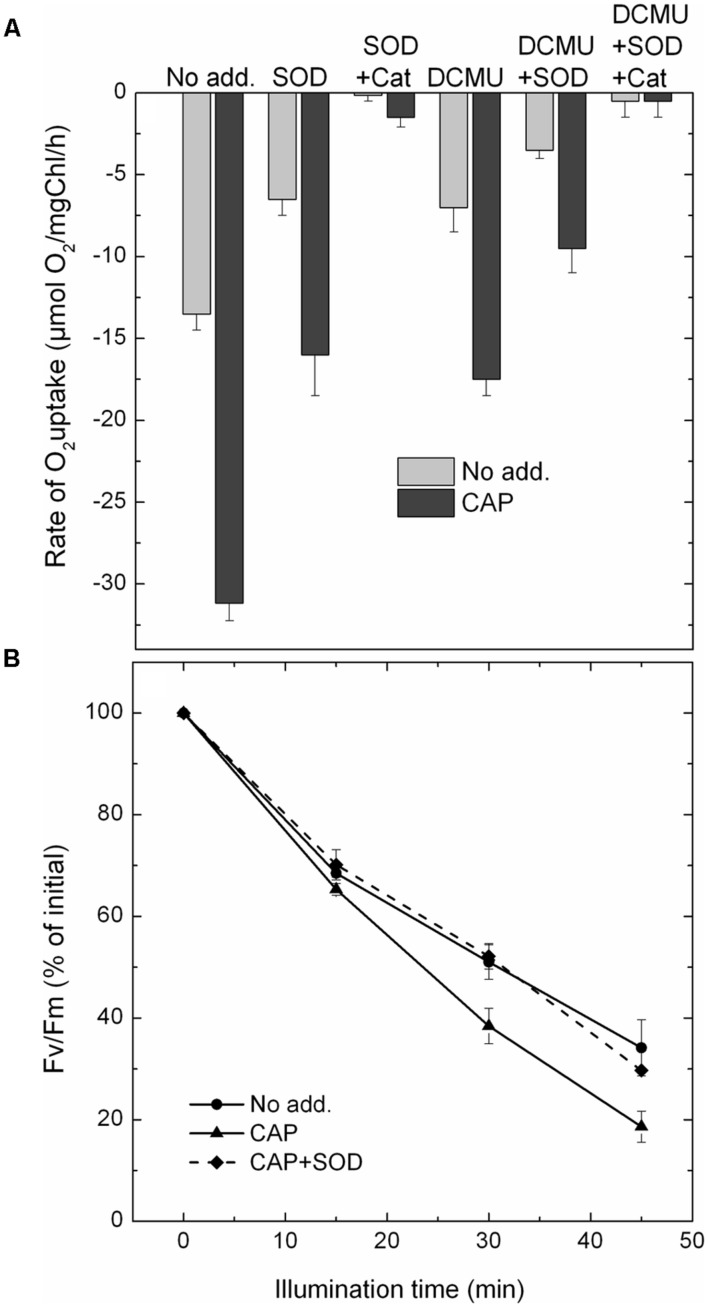
**The effect of CAP on superoxide production and photodamage in PSII.** **(A)** CAP mediated oxygen uptake. PSII membrane particles were illuminated, with 500 μmole m^-2^s^-1^ light intensity, in the chamber of a Clark-type electrode. The rate of O_2_ uptake was recorded in the presence and absence of CAP without further addition, in the presence of superoxide dismutase (SOD), and SOD + catalase. Similar series of experiments were also performed in the presence of the electron transport inhibitor DCMU. **(B)** CAP mediated photodamage. PSII membrane particles were exposed to illumination with 500 μmole m^-2^s^-1^ light intensity without addition, in the presence of CAP and CAP + SOD, and the activity of PSII was monitored by measuring variable Chl fluorescence (Fv/Fm).

In contrast to molecular oxygen superoxide does not produce amperometric signal in Clark-type oxygen electrodes. Therefore, conversion of O_2_ to 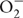 leads to oxygen consumption, which can be easily followed by oxygen uptake measurements. In order to investigate CAP mediated superoxide production we measured O_2_ uptake under various conditions. The data in **Figure 2A** show that CAP enhances light induced O_2_ uptake in PSII particles. This effect is partly reversible by SOD, which converts 1 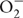 molecule to ½O_2_ and ½H_2_O_2_. Addition of catalase together with SOD almost completely eliminated the O_2_ uptake, which is consistent with the conversion of ½ H_2_O_2_ to ½ H_2_O + ½ O_2_. These data demonstrate that CAP can indeed mediate superoxide production in PSII. DCMU had only a minor inhibitory effect on the O_2_ uptake, which is consistent with the idea that CAP transfers electrons to oxygen from a PSII acceptor located before the Q_B_ site.

### CAP Enhances Photodamage of PSII in Isolated Membrane Particles

In order to check if CAP mediated superoxide production has any influence on the rate of photodamage PSII membrane particles were exposed to high light treatment in the absence and presence of CAP. According to the data shown in **Figure 2B**, light induced loss of PSII activity, as assessed by variable Chl fluorescence measurements, occurred faster in the presence than in the absence of CAP. Interestingly, the CAP induced enhancement of PSII photodamage was almost completely reversed when SOD was added together with CAP during the photoinhibitory treatment (**Figure 2B**). These data demonstrate that CAP induces enhanced PSII photodamage via production of superoxide in BBY particles.

## Conclusion

Our data show that CAP accepts electrons from the PSII complex at a site located before the Q_B_ quinone electron acceptor, most likely from Phe^-^. This process leads to superoxide production, which induces enhanced photodamage of PSII in isolated membrane particles. This side effect of CAP has potentially important implications regarding its application as protein synthesis inhibitor in photoinhibitory studies. Besides blocking the repair cycle of PSII CAP may accelerate the rate of photodamage also in intact systems leading to artifacts concerning the mechanism of photoinhibition. The *in vivo* effects of CAP are currently under investigation and will be presented in a forthcoming publication.

## Author Contributions

IV proposed the research topic, designed part of the experiments and contributed to writing of the manuscript. AUR, designed part of the experiments and contributed to data analysis and writing of the manuscript. SK performed most of the experiments and prepared the figures.

## Conflict of Interest Statement

The authors declare that the research was conducted in the absence of any commercial or financial relationships that could be construed as a potential conflict of interest.
